# Actor-partner effects associated with experiencing intimate partner violence or coercion among male couples enrolled in an HIV prevention trial

**DOI:** 10.1186/1471-2458-14-209

**Published:** 2014-02-28

**Authors:** Kristin M Wall, Patrick S Sullivan, David Kleinbaum, Rob Stephenson

**Affiliations:** 1 Department of Epidemiology, Rollins School of Public Health and Laney Graduate School, Emory University, 1518 Clifton Road NE, 4th Floor, Atlanta, GA 30322, Georgia; 2 Hubert Department of Global Health, Rollins School of Public Health and Laney Graduate School, Emory University, Atlanta, Georgia

**Keywords:** Actor-partner interdependence model, Coercion, HIV, Intimate partner violence, MSM

## Abstract

**Background:**

Intimate partner violence (IPV) and coercion have been associated with negative health outcomes, including increased HIV risk behaviors, among men who have sex with men (MSM). This is the first study to describe the prevalence and factors associated with experiencing IPV or coercion among US MSM dyads using the actor-partner interdependence model (APIM), an analytic framework to describe interdependent outcomes within dyads.

**Methods:**

Among MSM couples enrolled as dyads in an HIV prevention randomized controlled trial (RCT), two outcomes are examined in this cross-sectional analysis: 1) the actor experiencing physical or sexual IPV from the study partner in the past 3-months and 2) the actor feeling coerced to participate in the RCT by the study partner. Two multilevel APIM logistic regression models evaluated the association between each outcome and actor, partner, and dyad-level factors.

**Results:**

Of 190 individuals (95 MSM couples), 14 reported experiencing physical or sexual IPV from their study partner in the past 3 months (7.3%) and 12 reported feeling coerced to participate in the RCT by their study partner (6.3%). Results of multivariate APIM analyses indicated that reporting experienced IPV was associated (p < 0.1) with non-Black/African American actor race, lower actor education, and lower partner education. Reporting experienced coercion was associated (p < 0.1) with younger actor age and lower partner education.

**Conclusions:**

These findings from an HIV prevention RCT for MSM show considerable levels of IPV experienced in the past 3-months and coercion to participate in the research study, indicating the need for screening tools and support services for these behaviors. The identification of factors associated with IPV and coercion demonstrate the importance of considering actor and partner effects, as well as dyadic-level effects, to improve development of screening tools and support services for these outcomes.

## Background

Of the roughly 50,000 new HIV infections occurring annually in the United States (US), CDC estimates 61% occur among men who have sex with men (MSM), a group that accounts for 2% of the US population [1,2]. Due to disproportionally high HIV incidence among US MSM and the significantly increasing incidence rates observed among young, black MSM, MSM are an important focus of CDCs High-Impact Prevention approach to HIV prevention [2-4]. Additionally, given that an estimated 68% of new transmissions among MSM occur in the context of main partnerships [5], more prevention efforts are focusing on male couples as a prevention point [6-10].

Recent studies have also shown MSM to be at increased risk of experiencing intimate partner violence (IPV) relative to other men, at similar or higher rates compared to heterosexual females, though varying operational definitions of the numerous types of IPV and the different study recall periods make comparisons difficult [11-13]. The National Violence Against Women Survey (NVAWS) defines physical IPV as physical attacks or threats of attacks within a relationship. NVAWS defines sexual IPV as oral, anal, or vaginal penetration completed or attempted through force or threat of force [14]. A nationally representative probability sample of 14,182 participants of the NVAWS estimated that physical IPV experienced during any past or current relationship occurred among 25% of MSM, 8% of heterosexual males, and 21% of heterosexual females. This study estimated that experiences of sexual IPV occurred among 3% of MSM, 0.2% of heterosexual males, and 5% of heterosexual females [12]. These estimates are comparable with a probability-sample of 2,881 MSM from four US urban centers which found 22% of men experienced physical IPV (defined as being hit, pushed, shoved, kicked, or having something thrown at him) in the past 5 years, and 5% experienced sexual IPV (defined as being forced to have sex) in the past 5 years [13].

The reporting of experienced IPV by MSM has been strongly correlated with HIV/STI risk and more generally sexual risk-taking. Particularly, reporting experiencing of any form of IPV [15-18], of physical IPV [18], of sexual IPV [18,19], and of psychological/emotional IPV [20] is correlated with recent unprotected anal intercourse (UAI). This underscores the potential role of IPV as a significant risk factor for HIV among MSM.

Estimates of coercion, broadly defined as attempting to control the thoughts/behaviors of others, are scarcer and operational definitions more varied. Some studies include coercive control in the definition of IPV, and current research indicates coercion may be a precursor to IPV [21,22]. The NVAWS defined controlling IPV as attempts to control the actions or thoughts of a partner and found estimates of lifetime experienced controlling IPV of 82% among MSM and 41% among both heterosexual men and women [12].

A limited number of cross-sectional studies using various recall periods for the definition of IPV have identified factors, primarily demographics and negative health correlates, associated with IPV experienced by MSM. A cross-sectional survey of 817 MSM in Chicago found lifetime experienced IPV (sexual, physical, or verbal) was associated with frequent (monthly or more often) alcohol intoxication, substance abuse, receptive or insertive UAI in the past 6 months, increased sero-discordant UAI, sexually transmitted infection (STI) diagnoses in the previous 2 years, depression, and lifetime mental health diagnoses [15]. A study among 521 South African MSM showed that experiencing physical IPV in the past year was associated with non-white race, higher levels of education, and reporting receptive or insertive UAI in the past year, while experiencing sexual IPV in the past year was associated with experiences of homophobia [23]. Another study among 2,881 US MSM reported physical IPV experienced in the past 5 years was associated with younger age, being HIV positive versus negative, and lower education, while sexual IPV experienced in the past 5 years was associated with younger age [13]. A multinational study of 2,368 gay men from six countries found that, while demographic characteristics associated with IPV varied widely across countries, reporting homophobic discrimination was associated with experienced physical or sexual IPV in the past 12 months in all countries [24]. Finally, in one of the first studies to examine dyadic-level characteristics and IPV, data from an online survey of 528 US MSM couples showed men reporting non-white race and decreased relationship satisfaction were more likely to report physical IPV experienced with their study partner. Men reporting lower education, HIV positive serostatus (positive for anti-HIV antibodies), and decreased perceived stigma about having a male partner were more likely to report sexual IPV experienced with their study partner [10].

We aim to add to this body of literature by describing the prevalence of experienced IPV and coercion to participate in a research study among MSM dyads enrolled in an HIV prevention study. One aspect of coercion, generally defined as behavioral or mental coercive control [12], is coercion to participate in research or obtain health services. We hypothesized that the prevalence of IPV and coercion would be similar to heterosexual women, and that education would be associated with IPV.

We evaluated the association between demographic factors and these outcomes using the actor-partner interdependence model (APIM). The APIM is an analytic framework to describe interdependent outcomes within dyads. In this model, an outcome for an individual within the dyad is measured in terms of their actor (self) characteristics, partner characteristics, and their dyadic-level (shared) characteristics [25,26]. The APIM in an important tool for studying dyadic relationships and important outcomes that are influenced by the characteristics and actions of different members of a dyad, as well as measurements of their relationship to each other. Actor-partner effects among MSM have been evaluated for various health outcomes related to HIV risk including UAI within and outside the relationship [27], agreements about sex outside the relationship [28], and main and casual partner selection related to sero-sorting [7]. However, the actor-partner characteristics associated with IPV and coercion have yet to be studied among MSM.

## Methods

### Recruitment and eligibility

Male couples were recruited from the Atlanta area between 2010–2011 into a study of couples’ voluntary HIV counseling and testing (CVCT) as described elsewhere [29,30]. Briefly, eligible couples were at least 18 years old, had been in a partnership for at least 3 months, reported willingness to complete a 3 month follow-up survey, had never received a diagnosis of HIV, and could complete study assessments in English. Eligible participants provided informed consent and were given $50 for participation in the baseline survey and counseling session. This study was approved by Emory’s Institutional Review Board.

### Study procedures

Eligible and consenting participants separately answered a computer-administered baseline survey that collected demographic and couple characteristics, HIV testing history, sexual history, and several scales to measure aspects of couple functioning [6]. A survey administrator ensured that couples completed the survey at a sufficient physical distance so as to not influence one another. Two survey questions served as exclusionary criteria for randomization: history of experienced IPV (sexual or physical) in the past three months and feeling coerced by the study partner to test together. Couples in which either partner reported these exclusionary criteria were not considered eligible for randomization. These couples were informed that they would receive individual testing and were not told that one or both partners had reported IPV or coercion due to safety concerns.

### Exposures

Exposures of interest in this analysis included individual (both actor and partner) level demographic variables (age, race/ethnicity, education, sexual orientation, serostatus, UAI with a man other than (and concurrent with) the main study partner in the past 3 months, and agreements about sex outside the relationship. Dyad-level relationship variables included duration of relationship (calculated as the average reported by both partners), UAI in past year with the main partner (reported by either partner), and dyadic differences in age, education, race, sexual orientation, and sexual relationship agreements. The arms of the RCT were not of primary interest in this analysis, and for our purposes the data are cross-sectional.

### Outcome variables

Two primary outcomes were considered: 1) reporting a 3-month history of experienced IPV, either sexual or physical, from the study partner and 2) reporting feeling coerced to participate in the RCT by the study partner. History of IPV was measured using the following questions which were modified from the Conflict Tactics Scale [31]: “*In the past 3 months, has ___ hit you, kicked you, or physically hurt you?*” and “*In the past 3 months has ___ ever used force (hitting, holding down, or using a weapon) to make you have oral or anal sex?*” Coercion by the study partner to participate in the RCT was measured using the following question: “*Do you feel like ___ forced you to participate in this research study?*”

### Analyses

To describe individual-level and dyadic-level exposures, counts and percentages for categorical exposure variables and medians and interquartile ranges (IQRs) for continuous variables were tabulated separately for the outcomes of interest. Two-sided p-values from Chi-square tests (or Fisher’s Exacts tests) for categorical variables or median two-sample tests for continuous variables evaluated differences in individual-level and dyadic-level exposures separately for IPV and coercion. These descriptive analyses did not consider actor-partner effects.

**Figure 1:**
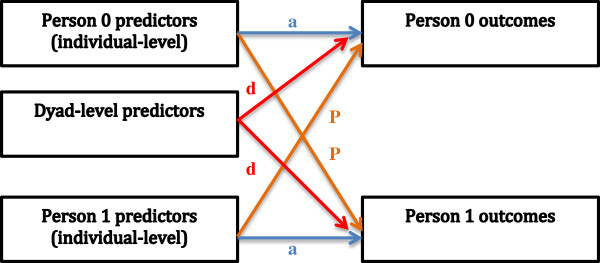
**Schematic of the actor-partner interdependence model (APIM) framework, adapted from Cook & Kenny, 2005 **[32]**. a,** actor effects. **p,** partner effects. **d,** dyad-level effects. Within-dyad residual errors between outcomes and predictors are not shown. **Individual-level predictors (actor and partner)**: Age, race/ethnicity, education, sexual orientation, serostatus, unprotected anal intercourse with a man other than main partner in past 3 months, and agreements about sex outside the relationship. **Dyad-level predictors:** Duration of relationship with main partner (average of partner responses); unprotected anal intercourse in last year with main partner (reported by either partner); dyadic differences in age, education, race/ethnicity, sexual orientation, and agreements about sex outside the relationship. **Outcomes:** 1) IPV experienced from the study partner in the past three months, 2) coercion experienced from the study partner to participate in the study.

The hypothesized association between actor, partner, and dyadic-level effects and each outcome is depicted schematically in Figure 1, adapted from Cook and Kenny [32]. Though not explicitly depicted in this schematic, dyads in this analysis are considered indistinguishable, meaning there is no meaningful way to distinguish outcomes between individual members of the dyad [25,26].

To determine the actor-partner and dyadic-level effects associated with each outcome, the dataset was structured in a pairwise format [25,26]. Continuous variables were grand-mean centered and binary categorical variables were dummy coded. The pairwise intra-class correlation coefficient (PICC), a measure of the extent of dyadic interdependence, was calculated for each outcome. Multi-level APIM models were specified for this analysis as shown in Table 1.

**Table 1:** Specification of the multi-level actor-partner interdependence model

**Levels**	**Predictor labels**	**Predictors**
Individual actor and individual partner (i)	X_actor, X_partner	Age, race/ethnicity, education, sexual orientation, serostatus, unprotected anal intercourse with a man other than main partner in past 3 months, agreements about sex outside the relationship
Dyad (j)	Z	Duration of relationship with main partner (average); unprotected anal intercourse in last year with main partner; dyadic differences in age, education, race/ethnicity, sexual orientation, and agreements about sex outside the relationship
**1. Individual level model:** g^‒ 1^(m_ij_) = **η**_**ij**_ = **β**_**0j**_ + **β**_**1j**_**(X** _ **actor)**_**ij**_ + **β**_**2j**_**(X** _ **partner)**_**ij**_		
Individual level residual term is omitted because its variance is assumed fixed		
**η**_**ij**_ is the log odds of the outcome		
**β**_**0j**_ is the within-dyad intercept in dyad j		
**β**_**1j**_ is the slope of η_ij_ on x_ij_ in dyad j		
**2. Dyad level model: β**_**0j**_ = **γ**_**00**_ + **γ**_**01**_**(Z)**_**j**_ + **u**_**oj**,_ **β**_**1j**_ = **γ**_**10**_, **β**_**2j**_ = **γ**_**20**_		
Dyad level slopes are fixed		
**u**_**0j**_**,** the random intercept, is the only random effect		
**γ**_**00**_ is the average intercept across dyads		
**3. Final model: η**_**ij**_ = **γ**_**00**_ + **γ**_**01**_**(Z)**_**j**_ + **γ**_**10**_**(X** _ **actor)**_**ij**_ + **γ**_**20**_**(X** _ **partner)**_**ij**_ + **u**_**oj**,_		
This model contains one random intercept (no random slopes, no interaction terms)		

Next, actor, partner, and dyadic-level effects were estimated following the 3-step multilevel modeling procedures for binary outcomes using an APIM framework as specified by McMahon et al., [33]. Briefly, PROC GENMOD was used to obtain initial values for the intercept and slope parameters using a generalized estimating equations approach (Appendix A1), PROC MIXED was used to determine initial values for the between-dyad variance (Appendix A2), and PROC NLMIXED was used to evaluate the random intercept model using these initial values (Appendix A3) (modified from McMahon et al., [33]).

The various analytical options used by McMahon et al., [33] were also utilized. The QPOINTS option defines the number of quadrature points needed for model convergence. The TECH = NEWRAP option stipulates that the Newton–Raphson algorithm is used as the parameter estimation optimization technique. The PARMS statement specifies the values of the beta parameters and the variance of the random effects obtained from PROC GENMOD and PROC MIXED. We also performed data analysis using an analogous 1-step PROC GLIMMIX procedure as described in Flom et al. [34] for comparison (Appendix A4). To build the multivariate APIM models of reported IPV and coercion, the above procedures were first used to estimate the independent (crude bivariate) associations between actor, partner, and dyadic-level factors and the outcomes of interest. Multivariate models were then built using backward selection procedures (using a cutoff of p < 0.1 for these exploratory analyses) to a model initially containing all exposures. Variables that were candidates for inclusion in the models were evaluated for multi-collinearity (cutoffs for multi-collinarity were taken as condition indices >30 and variance decomposition proportions >0.5). Odds ratios (OR) and 95% confidence intervals (95%CI) were calculated for all models. Data analysis was conducted with SAS v9.3 (Cary, NC).

## Results

### Individual-level factors independently associated with IPV and coercion

In this study of 190 individuals (95 couples), 14 individuals reported experiencing physical or sexual IPV from their study partner in the past 3 months (7.3%). There were 12 individual reports of experienced physical IPV and 4 individual reports of experienced sexual IPV, with two individuals experiencing both behaviors. Twelve individuals reported experiencing coercion (6.3%). Individuals from two couples reported experiencing both IPV and coercion. Within one couple, both partners experienced coercion. The magnitude and direction of the associations between white/Caucasian race (n = 30) and the outcomes, and other races (n = 19) and the outcomes, were similar. These race categories were grouped (n = 49) for analysis due to small numbers among the coercion outcome.

In bivariate analyses, individuals reporting IPV were older on median than individuals not reporting IPV (33.5 years versus 30.0 years, p = 0.01). Individuals who reported having a high school/GED education or less were more likely to report IPV relative to individuals with some post-high school education (13% versus 2%, p = 0.004) (Table 2).

**Table 2:** Individual-level demographic characteristics associated with experiencing IPV or coercion

	**Total (N = 190)**		**No experienced IPV (N = 176)**		**Experienced IPV (N = 14)**		p-value*	**No experienced coercion (N = 178)**		**Experienced coercion (N = 12)**		p-value*
	**N**	**col%**	**N**	**row%**	**N**	**row%**		**N**	**row%**	**N**	**row%**	
**Age, median, IQR (years)**	30.0	14.0	30.0	15.0	33.5	12.0	0.01	30.0	15.0	28.0	15.0	0.406
**Race/ethnicity**							0.20					0.07
Black/African American	137	74%	129	94%	8	6%		126	92%	11	8%	
Other	49	26%	43	88%	6	12%		49	100%	0	0%	
**Education**							0.004					0.48
Some education post-high school	98	52%	96	98%	2	2%		93	95%	5	5%	
High school, GED, or less	92	48%	80	87%	12	13%		85	92%	7	8%	
**Sexual orientation**							1.00					0.36
Homosexual/gay	116	63%	108	93%	8	7%		110	95%	6	5%	
Bisexual/other	68	37%	63	93%	5	7%		62	91%	6	9%	
**Serostatus**							1.00					1.00
HIV positive	20	11%	19	95%	1	5%		19	95%	1	5%	
HIV negative	170	89%	157	92%	13	8%		159	94%	11	6%	
**UAI with an outside (and concurrent with main) partner in past 3 months**							1.00					0.46
Yes	38	21%	36	95%	2	5%		37	97%	1	3%	
No	145	79%	135	93%	10	7%		135	93%	10	7%	
**Agreements about sex outside the relationship**							0.12					0.42
Monogamy	105	56%	100	95%	5	5%		97	92%	8	8%	
Other (Outside sex, no agreement)	84	44%	75	89%	9	11%		80	95%	4	5%	

*Two-sided p-values from Chi-square tests or Fisher’s Exacts tests (categorical variables) or Median two-sample tests (continuous variables).

Cells may not add to total due to missing values.

GED: general educational development; IPV: intimate partner violence; IQR: interquartile range; UAI: unprotected anal intercourse.

### Dyad-level factors independently associated with IPV and coercion

In bivariate analyses, couples reporting coercion had a relatively larger dyadic-difference in median age (6.0 years versus 4.0 years, p = 0.02) (Table 3).

**Table 3:** Dyad-level demographic characteristics associated with experiencing IPV or coercion

	**Total (N = 95)**		**No experienced IPV (N = 81)**		**Experienced IPV (N = 14)**		p-value*	**No experienced coercion (N = 84)**		**Experienced coercion (N = 11)**		p-value*
	**N**	**col%**	**N**	**row%**	**N**	**row%**		**N**	**row%**	**N**	**row%**	
**Duration of relationship with main partner, median, IQR (months) (average reported by both partners)**	14.0	17.3	13.4	16.0	22.1	21.7	0.08	13.9	17.0	14.4	24.5	0.35
**UAI with main partner in past year (reported by either partner)**							0.53					0.73
Yes	58	65%	48	83%	10	17%		52	90%	6	10%	
No	31	35%	28	90%	3	10%		27	87%	4	13%	
**Difference in age, median, IQR (years)**	4.0	6.0	4.0	6.0	4.5	4.0	0.97	4.0	5.5	6.0	6.0	0.02
**Difference in education**							0.34					1.00
Report same	69	73%	57	83%	12	17%		61	88%	8	12%	
Report different	26	27%	24	92%	2	8%		23	88%	3	12%	
**Difference in race**							1.00					0.67
Report same (both black, white, other)	76	84%	64	84%	12	16%		68	89%	8	11%	
Report different	15	16%	13	87%	2	13%		13	87%	2	13%	
**Difference in orientation**							0.35					1.00
Report same	56	63%	50	89%	6	11%		49	88%	7	13%	
Report different	33	37%	27	82%	6	18%		29	88%	4	12%	
**Difference in agreements**							0.80					0.46
Report same	50	53%	43	86%	7	14%		43	86%	7	14%	
Report different	44	47%	37	84%	7	16%		40	91%	4	9%	

Cells may not add to total due to missing values.

IPV: intimate partner violence; IQR: interquartile range; UAI: unprotected anal intercourse.

*Two-sided p-values from Chi-square tests or Fisher’s Exacts tests (categorical variables) or Median two-sample tests (continuous variables).

### Actor-partner and dyad-level factors associated with IPV

The estimates obtained from implementing the 3-step analysis method described (PROC GENMOD, MIXED, and NLMIXED) were very similar to those obtained using the 1-step PROC GLIMMIX procedure for all models. The 3-step method results are presented for all analyses as this method produces an approximation to the likelihood with a log-likelihood fit statistic and is thought to produce more valid results [34].

In multivariate analysis, non-black/African American actor race (p = 0.02), actor high school/GED education or less (p = 0.06), and partner high school/GED education or less (p = 0.06) were associated with experiencing IPV (Table 4). No collinearity was detected between model variables and no significant interaction terms were discovered.

**Table 4:** Actor-partner interdependence model of factors associated with experiencing IPV

	**Crude OR**	**(90% CI)**		**p-value**	**Adjusted OR**	**(90% CI)**		**p-value**
**ACTOR VARIABLES**								
**Age, per year increase**	1.05	1.00	1.09	0.09				
**Race/ethnicity**								
Black/African American	ref							
Other	2.25	0.88	5.77	0.16	4.25	1.49	12.12	0.024
**Education**								
Some education post-high school	ref							
High school, GED, or less	7.20	1.98	26.18	0.013	5.01	1.23	20.45	0.06
**Sexual orientation**								
Homosexual/gay	ref							
Bisexual/other	1.07	0.40	2.86	0.91				
**Serostatus**								
HIV positive	ref							
HIV negative	1.57	0.27	9.21	0.67				
**UAI with a man other than (and concurrent with) main partner in past 3 months**								
Yes	ref							
No	1.33	0.36	5.00	0.72				
**Agreements about sex outside the relationship**								
Monogamy	ref							
Other (Outside sex, no agreements)	2.40	0.92	6.26	0.13				
**PARTNER VARIABLES**								
**Age, per year increase**	1.05	1.01	1.10	0.05				
**Race/ethnicity**								
Black/African American	ref							
Other	1.13	0.41	3.14	0.85				
**Education**								
Some education post-high school	ref							
High school, GED, or less	7.20	1.98	26.18	0.01	5.14	1.26	20.92	0.056
**Sexual orientation**								
Homosexual/gay	ref							
Bisexual/other	1.51	0.58	3.93	0.48				
**Serostatus**								
HIV positive	ref							
HIV negative	1.57	0.27	9.21	0.67				
**UAI with a man other than (and concurrent with) main partner in past 3 months**								
Yes	1.78	0.62	5.06	0.36				
No	ref							
**Agreements about sex outside the relationship**								
Monogamy	ref							
Other (Outside sex, no agreements)	1.74	0.68	4.40	0.33				
**DYAD-LEVEL VARIABLES**								
**Duration of relationship with main partner (average reported by both partners, per year increase)**	1.01	1.00	1.03	0.15				
**UAI with main partner in past year (reported by either partner)**								
Yes	1.86	0.60	5.71	0.36				
No	ref							
**Difference in age (per year decrease)**	1.02	0.93	1.13	0.69				
**Difference in education**								
Report same	2.36	0.65	8.70	0.28				
Report different	ref							
**Difference in race**								
Report same (both black, white, other)	1.20	0.32	4.46	0.82				
Report different	ref							
**Difference in orientation**								
Report same	ref							
Report different	1.77	0.65	4.78	0.35				
**Difference in agreements**								
Report same	ref							
Report different	1.15	0.46	2.89	0.80				

GED: general educational development; IPV: intimate partner violence; UAI: unprotected anal intercourse.

### Actor-partner and dyad-level factors associated with coercion

In multivariate analysis, younger actor age (p = 0.098) and partner high school/GED education or less (p = 0.09) were associated were associated with experiencing coercion (Table 5). No collinearity was detected between model variables and no significant interaction terms were discovered.

**Table 5:** Actor-partner interdependence model of factors associated with experiencing coercion

	**Crude OR**	**(90% CI)**		**p-value**	**Adjusted OR**	**(90% CI)**		**p-value**
**ACTOR VARIABLES**								
**Age, per year decrease**	1.05	0.99	1.11	0.21	1.06	1.00	1.13	0.098
**Race/ethnicity**								
Black/African American	n/a							
Other								
**Education**								
Some education post-high school	ref							
High school, GED, or less	1.53	0.56	4.18	0.48				
**Sexual orientation**								
Homosexual/gay	ref							
Bisexual/other	1.77	0.66	4.79	0.34				
**Serostatus**								
HIV positive	ref							
HIV negative	1.31	0.22	7.78	0.80				
**UAI with a man other than (and concurrent with) main partner in past 3 months**								
Yes	2.74	0.47	16.05	0.35				
No	ref							
**Agreements about sex outside the relationship**								
Monogamy	1.65	0.58	4.70	0.43				
Other (Outside sex, no agreements)	ref							
**PARTNER VARIABLES**								
**Age, per year decrease**	1.02	0.97	1.07	0.57				
**Race/ethnicity**								
Black/African American	1.85	0.50	6.90	0.44				
Other	ref							
**Education**								
Some education post-high school	ref							
High school, GED, or less	2.24	0.79	6.37	0.21	3.04	1.03	9.00	0.092
**Sexual orientation**								
Homosexual/gay	ref							
Bisexual/other	1.77	0.66	4.79	0.34				
**Serostatus**								
HIV positive	1.78	0.46	6.86	0.48				
HIV negative	ref							
**UAI with a man other than (and concurrent with) main partner in past 3 months**								
Yes	1.19	0.31	4.52	0.83				
No	ref							
**Agreements about sex outside the relationship**								
Monogamy	1.13	0.41	3.08	0.84				
Other (Outside sex, no agreements)	ref							
**DYAD-LEVEL VARIABLES**								
**Duration of relationship with main partner (average reported by both partners, per year increase)**	1.01	1.00	1.03	0.46				
**UAI with main partner in past year (reported by either partner)**								
Yes	ref							
No	1.61	0.57	4.56	0.45				
**Difference in age (per year increase)**	1.01	0.92	1.11	0.87				
**Difference in education**								
Report same	1.13	0.36	3.56	0.86				
Report different	ref							
**Difference in race**								
Report same (both black, white, other)	ref							
Report different	1.14	0.30	4.34	0.88				
**Difference in orientation**								
Report same	1.19	0.42	3.41	0.78				
Report different	ref							
**Difference in agreements**								
Report same	1.25	0.46	3.41	0.71				
Report different	ref							

GED: general educational development; UAI: unprotected anal intercourse.

## Discussion

In this analysis of MSM participating in an HIV prevention study as dyads, our estimates of physical or sexual IPV in the previous 3 months (7% prevalence) and experienced coercion to participate in the study (6% prevalence) were similar to studies measuring more recent IPV among MSM. A study of MSM from 6 countries found 5.8% of US MSM reported experiencing physical IPV in the past year, and 4.5% reported experiencing sexual IPV in the past year [24]. To our knowledge, there are no large population-based estimates of coercion as defined here among MSM dyads.

There are currently no other published studies examining both the actor-partner effects in addition to the shared dyad-level characteristics associated with these outcomes among MSM. Evaluating actor-partner effects within the APIM framework is advantageous because it considers how one partner’s exposures may influence the other partner’s outcomes. These nuanced associations can be missed when looking at data at the individual-level only. For example, race was not significantly associated with experiencing IPV at the individual-level but was a significant actor effect in the multivariate actor-partner model.

### Actor-partner effects associated with experienced IPV

The actor reporting non-black/African American race was associated with experiencing IPV in the past 3 months relative to the actor being black/African American. Since our sample was predominately black/African American, we were not able to evaluate race differences in more depth. The existing literature regarding race and IPV among MSM is varied and conflicting – for example, in the previously described study of 528 US MSM, non-white race was found to be significantly associated with experiencing physical IPV [10], while the results of the study of 2,881 US MSM indicated that race was not associated with reporting physical or sexual IPV [13]. These differences highlight a recurring theme in the current literature: demographic characteristics hypothesized to be associated with IPV do not translate to every MSM population, especially in the multinational study by Finnernan et al. [24]. For example, we did not find an association between age and experiences of recent IPV, and again results are varied in the current literature – some studies indicate younger age is associated with experienced physical or sexual IPV among MSM [13,24], while other studies among MSM observed no association with age [23,35]. Younger age is a classic risk factor for IPV experienced by heterosexual women, seemingly linked to the fact men tend to become less violent with age [36], but this association does not appear consistent across MSM populations.

Lower education was associated with reporting experienced IPV in the past 3 months in this study. Many investigations indicate a link between lower education and violence among MSM [10,13,35], as lower education may be associated with decreased access to economic, social, and health resources thereby increasing vulnerability. However, in the current study we further show that both actor and partner educational level have an effect.

### Actor-partner effects associated with experienced coercion

The actor-partner and dyadic-level factors associated with experiencing coercion to participate in the study were younger actor age and lower partner education. While we are unaware of similar investigations of factors associated with coercion to participate in research studies between MSM couples for comparison, younger age and lower education have been associated with controlling IPV [12], and we hypothesize that the younger age of the actor creates a power dynamic making them more susceptible to experiencing coercive control. A study by Greenwood et al. [13], which found a role for age in all forms of IPV, hypothesized that older persons may be better able to access resources and protection’s than younger people, or that younger people may be easier to influence [13].

### Screening for IPV and coercion in research and programmatic settings

IPV and coercion were screened for in this RCT in order to nonrandomly allocate couples reporting these behaviors to receive individual HIV testing, because the effect of the CVCT service on these behaviors is currently unclear. Additionally, in a research setting, coercion to participate in a study represents a threat to human subjects and bias study results. In a programmatic setting, screening for coercion to participate in programs designed for male-male couples is important to ensure that services are delivered to clients who both desire and have independently chosen to receive the service.

More generally, in couples-focused research or programmatic settings, screening for IPV among MSM couples is an important opportunity for referrals for IPV support services. Importantly, evidence suggests coercive control may be an upstream behavior leading to IPV [21,22] further motivating the rationale for screening for coercion in order to refer persons reporting this behavior for support services [22].

### Screening tool and support service development

IPV screening tools do not currently have well-established psychometric soundness for use among MSM, the sensitivities and specificities between current screening tools vary greatly, few consider coercion a separate behavior, and the most common screening tools have been evaluated in relatively few studies among primarily heterosexuals [21,22,37]. IPV support services for MSM are also inadequate -- awareness of these issues among MSM is low, many US domestic violence services do not serve men, and IPV victims from same-sex relationships are not provided civil protections in several states [12,38]. This preliminary understanding actor-partner effects and dyadic differences related to IPV and coercion suggests that screening tools and support services can benefit from an understanding of both actor and partner effects, and that they may benefit from targeting younger, less educated MSM. Though our findings are preliminary, we believe that other, larger studies designed to better understanding these associations should be conducted to inform the development of screening tools and support services.

### Limitations

Several limitations to this study warrant consideration. The small sample size and exploratory nature of our analysis (p < 0.1) did not allow for a deeper investigation of several associations, especially investigation of racial/ethnic differences and these outcomes, or the differences between physical and sexual IPV. Since IPV was not the primary study outcome, it was assessed using only two modified items from the Conflict Tactics Scale. If we had assessed IPV more comprehensively using the entire scale, we likely would have discovered more cases on IPV. Selection bias affecting who decided to participate in the study and who decided to answer the questions about IPV and coercion could limit the generalizability of these findings to MSM in couples who were interested in being tested together for HIV. Although measures were taken to ensure confidentiality in reporting IPV and coercion, misclassification of sensitive relationship outcomes is common [39,40] and may have affected the validity of our findings, likely underestimating the true prevalence of IPV and coercion. Additionally, given that these data are cross-sectional, we were only able to evaluate associations and not possible causal mechanisms. Longitudinal data exploring the causal associations between couple characteristics and IPV and coercion among MSM are needed.

Because this study was not designed or powered to detect differences in these outcomes, the analyses are exploratory and results should be interpreted with caution.

However, this study represents the first steps toward understanding the main actor-partner effects and dyadic characteristics related to IPV and coercion. This novel use of the APIM employing systematic model building techniques would benefit from being applied to larger sample sizes and more diverse populations of MSM couples with consideration of the frequency and severity of IPV and coercion. Other exposures associated with IPV and coercion in previous studies also need to be considered in terms of actor and partner effects including substance abuse [15,41], homophobic discrimination and internalized homophobia [23,24,35], experiences of homophobia [23,24], history of violence [35], relationship concurrency, and perceived stigma [10].

## Conclusions

Understanding the prevalence and factors associated with IPV and coercion among MSM increases awareness of these issues and the need for better screening tools and support services. Based on the results of this study and the current literature, we recommend future studies evaluate actor, partner, and dyadic-level predictors of IPV and coercion toward the goal of improving screening tools and support services for IPV and coercion among MSM couples.

## Appendix

A1. Obtain initial values for the intercept and slope parameters:

$$\begin{array}{l} \;\text{proc}\;\text{genmod}\;\text{data}=\text{dataset}\;\text{descending};\\ \;\text{class}\;\text{dyad};\\ \;\text{model}\;\text{outcome}=X\_\text{partner}\;X\_\text{actor}\;Z/\text{dist}=\text{bin}\;\text{link}=\mathrm{logit};\\ \;\text{repeated}\;\text{subject}=\text{dyad}/\text{type}=\mathrm{un};\\ \;\text{run}; \end{array}$$

A2. Determine initial values for the between-dyad variance:

$$\begin{array}{l} \;\text{proc}\;\text{mixed}\;\text{data}=\text{dataset}\;\text{method}=\text{reml};\\ \;\text{class}\;\text{dyad};\\ \;\text{model}\;\text{outcome}=X\_\text{partner}\;X\_\text{actor}\;Z/\text{solution};\\ \;\text{random}\;\text{intercept}/\text{subject}=\text{dyad};\\ \;\text{run}; \end{array}$$

A3. Evaluate the random intercept model using the initial values from steps A1 and A2:

$$\begin{array}{l} \;\text{proc}\;\text{nlmixed}\;\text{data}=\text{dataset}\;\text{qpoints}=20\;\text{tech}=\text{newrap};\\ \;\text{parms}\;\mathrm{beta}0=x0\;\mathrm{beta}1=x1\mathrm{beta}2\\ \;=x2\;\mathrm{beta}3=x3\;s2u=x4;\\ \;\mathrm{eta}=\mathrm{beta}0+\mathrm{beta}1*X\_\text{actor}\\ \;+\mathrm{beta}2*X\_\text{partner}+\mathrm{beta}3*Z+u;\\ \;\mathrm{mu}=\exp\left(\mathrm{eta}\right)/\left(1+\exp\;\left(\mathrm{eta}\right)\right);\\ \;\text{model}\;\text{outcome}\sim \text{binary}\;\left(\mathrm{mu}\right);\\ \;\text{random}\;u\sim \text{normal}\left(0,s2u\right)\text{subject}\\ \;=\text{dyad}\\ \;\text{run}; \end{array}$$

A4. One step procedure to obtain beta estimates:

$$\begin{array}{l} \;\text{proc}\;\text{glimmix}\;\text{data}=\text{dataset};\\ \;\text{class}\;\text{dyad};\\ \;\text{model}\;\text{outcome}=X\_\text{actor}\;X\_\text{parnter}\;Z\\ \;/\text{solution}\;\text{link}=\mathrm{logit}\;\text{dist}=\text{binomial};\\ \;\text{random}\;\text{intercept}/\text{subject}=\text{dyad}\;\text{type}\\ \;=\mathrm{un}\;\mathrm{gcorr};\\ \;\text{run}; \end{array}$$

## Abbreviations

APIM: Actor-partner interdependence model; CVCT: Couples’ voluntary HIV counseling and testing; IPV: Intimate partner violence; IQR: Interquartile range; MSM: Men who have sex with men; NVAWS: National violence against women survey; RCT: Randomized controlled trial; UAI: Unprotected anal intercourse.

## Competing interests

The authors declare that they have no competing interests.

## Authors’ contributions

KW made substantial contributions to the analysis and interpretation of data, drafting the article and revising it critically for important intellectual content, and gave final approval of the version to be published. PS made substantial contributions to study conception and design, acquisition of data, was involved in drafting the manuscript or revising it critically for important intellectual content, and gave final approval of the version to be published. DK made substantial contributions to the analysis and interpretation of data, revising the article critically for important intellectual content, and gave final approval of the version to be published. RS made substantial contributions to study conception and design, acquisition of data, was involved in drafting the manuscript or revising it critically for important intellectual content, and gave final approval of the version to be published. All authors read and approved the final manuscript.

## Pre-publication history

The pre-publication history for this paper can be accessed here:

http://www.biomedcentral.com/1471-2458/14/209/prepub
